# Hydrogen gas inhalation ameliorates cardiac remodelling and fibrosis by regulating NLRP3 inflammasome in myocardial infarction rats

**DOI:** 10.1111/jcmm.16863

**Published:** 2021-08-16

**Authors:** Chaoqun Nie, Rentong Zou, Shuang Pan, Rong A, Yunan Gao, Hongxiao Yang, Juncai Bai, Shuiqing Xi, Xue Wang, Xiaojian Hong, Wei Yang

**Affiliations:** ^1^ Department of Cardiology The Fourth Affiliated Hospital of Harbin Medical University Harbin China; ^2^ NHC and CAMS Key Laboratory of Molecular Probe and Targeted Theranostics Molecular Imaging Research Center (MIRC Harbin Medical University Harbin China

**Keywords:** cardiac remodelling, fibrosis, hydrogen, myocardial infarction, NLRP3 inflammasome, pyroptosis

## Abstract

It is noteworthy that prolonged cardiac structural changes and excessive fibrosis caused by myocardial infarction (MI) seriously interfere with the treatment of heart failure in clinical practice. Currently, there are no effective and practical means of either prevention or treatment. Thus, novel therapeutic approaches are critical for the long‐term quality of life of individuals with myocardial ischaemia. Herein, we aimed to explore the protective effect of H_2_, a novel gas signal molecule with anti‐oxidative stress and anti‐inflammatory effects, on cardiac remodelling and fibrosis in MI rats, and to explore its possible mechanism. First, we successfully established MI model rats, which were then exposed to H_2_ inhalation with 2% concentration for 28 days (3 hours/day). The results showed that hydrogen gas can significantly improve cardiac function and reduce the area of cardiac fibrosis. In vitro experiments further proved that H_2_ can reduce the hypoxia‐induced damage to cardiomyocytes and alleviate angiotensin II‐induced migration and activation of cardiac fibroblasts. In conclusion, herein, we illustrated for the first time that inhalation of H_2_ ameliorates myocardial infarction‐induced cardiac remodelling and fibrosis in MI rats and exert its protective effect mainly through inhibiting NLRP3‐mediated pyroptosis.

## 1 INTRODUCTION

Ischaemic heart disease is the most important cause of death in the world, and it is also the most common cause of heart failure.^1^ With the advancements of modern medical therapy approaches, a growing number of individuals with myocardial infarction are acceptable to receive revascularization therapy in time. Nevertheless, a large number of patients still suffer from myocardial ischaemia, mainly due to the failure of optimal surgical timing or unsuccessful surgical procedures.^2^ It is worth noting that cardiac remodelling and fibrosis are the important outcomes of prolonged myocardial infarction, which are closely related to the occurrence of heart failure.^3^, ^4^, ^5^ Thus, novel therapeutic approaches against cardiac remodelling and myocardial fibrosis are critical for the long‐term quality of life of patients with myocardial ischaemia.^6^

Hydrogen gas (H_2_) is a colourless and easily combustible gas at normal temperature and pressure. In the past, it was considered to be an inert gas without any biological effect.^7^ Ohta et al first discovered the selective anti‐oxidation mechanism of H_2_ for the treatment of ischaemia–reperfusion injury, which has emerged as the force behind the rapid developments of the application of H_2_ in biology and medicine.^8^ Most importantly, hydrogen, the smallest and lightest gaseous molecule, which easily diffuses in cells and tissues, has great potential for clinical translation because of its convenience, economy and especially high biosafety.^9^

Hydrogen has been proved to possess a significant effect on the inhibition of oxidative stress, anti‐apoptosis and anti‐inflammation.^10^ In the past decade, H_2_ was found to exhibit treatment effects on a variety of oxidative stress and inflammation‐linked diseases, consisting of stroke, cancer, diabetes, atherosclerosis, ischaemia–reperfusion injury and neurodegenerative diseases.^10^, ^11^, ^12^, ^13^, ^14^ However, the effect of H_2_ on long‐term cardiac remodelling and myocardial fibrosis in MI rats remains unknown.

The NLR family pyrin domain containing 3 (NLRP3) inflammasome components mainly include 3 members: NLRP3, ASC and Procaspase‐1.^15^ Mechanistically, activated NLRP3 inflammasome cleaves Gasdermin‐D (GSDMD) within its linking loop to release its autoinhibition on its GSDMD‐N domain, leading to the generation of plasma membrane pores and driving the release of IL‐1β along with IL‐18, which play a direct role in triggering inflammatory‐dependent cell death called pyroptosis.^16^, ^17^, ^18^ Reports have shown that inflammasome as a new player in myocardial infarction promotes adverse cardiac remodelling.^19^

In this study, we sought to reveal the protective effect of H_2_ on cardiac remodelling and myocardial fibrosis in vivo and in vitro, and to further explore the potential mechanism of biological effect of H_2_ on myocardial infarction.

## 2 MATERIALS AND METHODS

### 2.1 Animals and experimental protocol

Male Sprague Dawley (SD) rats weighing 150–180 g were acquired from animal experiment centre of Harbin Medical University (Harbin, China). The animals were bred and maintained under standard conditions (12 h light‐dark cycle, 24°C), with free access to standard laboratory chow, as well as water. Rats were weaned at postnatal day 21, and two to five rats were housed per cage. The Institutional Animal Care and Use Committee, Harbin Medical University, approved the experimental protocol involving the use of rats (Approval No. 20200915). Five experimental groups were randomly organized with fifteen rats each: the sham group, the myocardial infarction group (MI group), the myocardial infarction rat with hydrogen gas inhalation group (MI + H_2_ group), the myocardial infarction rat with the MCC950 group (MI + MCC950 group), the myocardial infarction rat with the MCC950 and hydrogen gas inhalation group (MI + MCC950 + H_2_ group). MCC950, a selective, potent, small‐molecule inhibitor of NLRP3 (CP‐456773, Selleck, USA), was used as a positive control. The main way of MCC950 is to prevent the hydrolysis of ATP, thus organizing the assembly of inflammatory bodies.^20^, ^21^

### 2.2 Rat myocardial infarction model

After 1 week of adaptation, rats with MI were established via permanent LCA (ligation of the left coronary artery). Briefly, rats were intubated and ventilated after being anaesthetized with 1% Pentobarbital solution (40 mg/kg), and buprenorphine (0.05 mg/kg) was injected intraperitoneally for perioperative analgesia. We performed left intercostal thoracotomy to partially expose the heart, and then, subsequent LCA ligation was done with a 6–0 nylon suture. The ischaemic state of rat heart was confirmed by evidence of immediate changes, including sudden pallor and paralysis of the affected part of the left ventricle with the ECG variation that ST‐T segment elevation displayed apparently. To prevent postoperative infection, rats were injected with 20000 units of penicillin intramuscularly for three consecutive days after myocardial infarction operation. After 28 days, each group rats were sampled. For Sham group, we passed the suture around LCA and removed it without ligation.

### 2.3 Inhalation of H_2_ and MCC950 administration protocol

**FIGURE 1:**
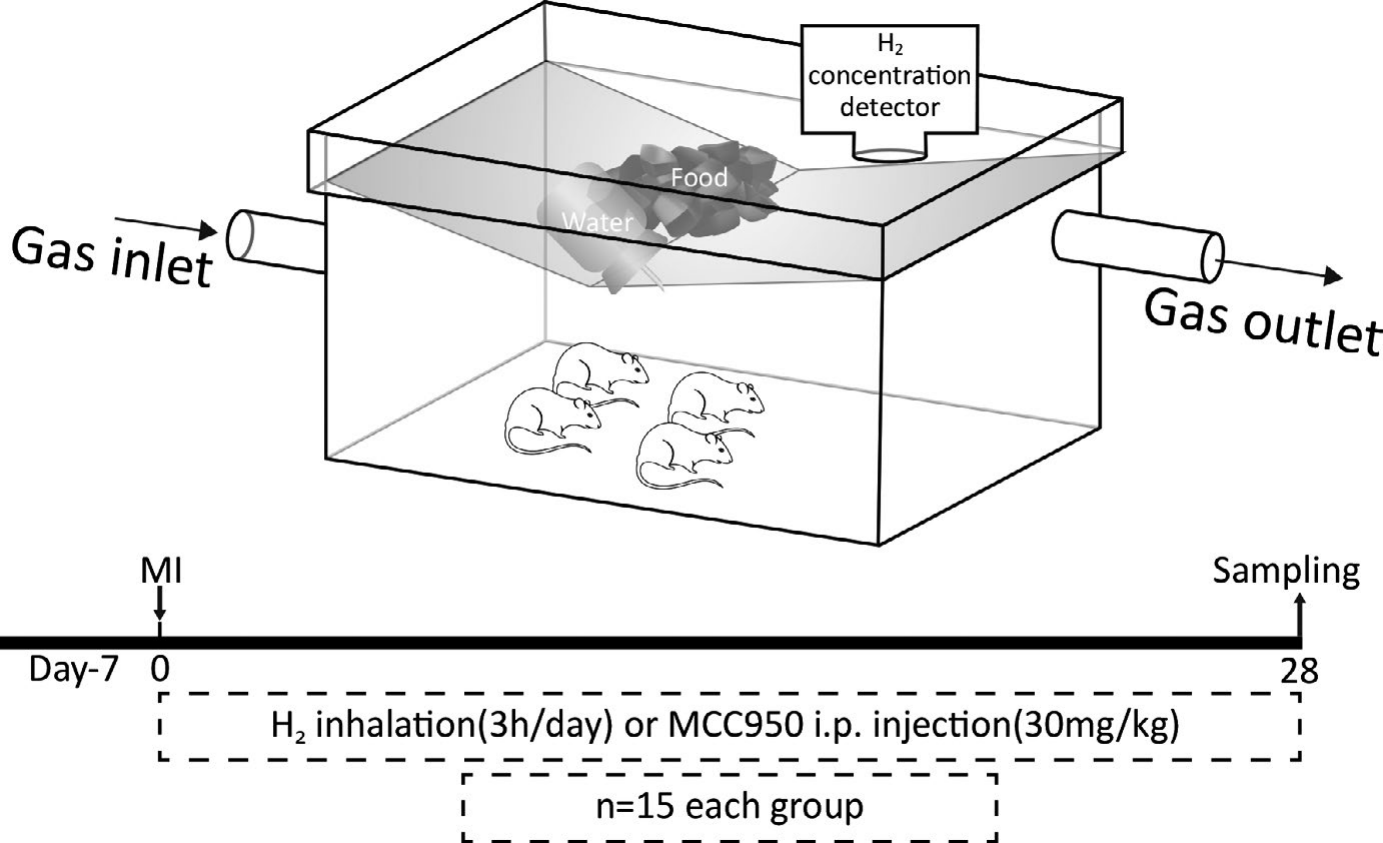
Schematic diagram of H_2_ inhalation and experimental protocol. The hydrogen inhalation device is specially designed to allow the stable hydrogen concentration through connecting the 2% H_2_ output pipe seamlessly. The hydrogen detector detects the internal hydrogen concentration real time. After successfully conducted MI model, the treatment group began to inhaled 2% hydrogen 3h /day and/or i.p. injection MCC950 30 mg/kg

Considering the unique physical and chemical properties of H_2_, including the ability to easily explode as well as burn under atmospheric levels of 4% to 75%, for high safety, we selected 2% H_2_ concentration to explore its therapeutic effect. The air pump generated air and the pore hydrogen gas generator (Dura Safer Technology, China) generated H_2_ were mixed together (with the ratio flow rates of 50:1 per minute) to obtain 2% H_2_. Real‐time detection of internal H_2_ concentration detection in a manmade rat cage was done using a seamlessly connected hydrogen concentration detector (RWD Biotech, China. H_2_ group rats were subjected to 2% H_2_ inhalation for 3 h/day. MCC950, a selective, potent, small‐molecule inhibitor of NLRP3 (CP‐456773, Selleck, USA) was dispersed in sterile saline. According to previous studies, the MI + MCC950 group and the MI + MCC950 + H_2_ group were injected MCC950 intraperitoneally at the recommended single dose of 30 mg/kg after MI surgery.^22^, ^23^ Sham group, MI group and MI + H_2_ group were administered with an equivalent volume of sterile saline as control (Figure 1).

### 2.4 Echocardiographic evaluation of cardiac function and structure

A Philips high‐resolution HD11XE ultrasound system with S12‐4 (4–12 MHz) probe (Philips Healthcare Ultrasound, Netherlands) was employed to perform echocardiography to assess the cardiac function of rat before kill. All echocardiographic evaluations were performed by cardiologists dedicated in the field of cardiac imaging. The parasternal short‐axis view in the left lateral decubitus position of the M‐mode echocardiogram provided the left ventricular function. Ejection fraction (EF) along with Fractional shortening (FS) assessments was conducted to examine the left ventricular (LV) systolic function of the heart. Besides, measurement of the left ventricular internal diameter at end‐diastole (LVIDd), as well as left ventricular internal diameter at end‐systole (LVIDs), was conducted to evaluate cardiac structural changes. The average of three successive cardiac cycles was utilized.

### 2.5 ELISA

The plasma used in ELISA was obtained aseptically from abdominal aorta and was collected into EDTA and centrifuged for 15 minutes at 1000 *g* within 30 minutes of collection. The concentration of BNP in plasma was measured using ELISA kit (Cusabio Bio, E07972r, China) as described by the manufacturer.

### 2.6 Transmission electron microscopy

Cardiac tissues were fixed with 2.5% glutaraldehyde and 1% OsO_4_, followed by embedment in resin. Counterstaining of ultra‐thin sections was performed using uranyl acetate and lead citrate. Lastly, a transmission electron microscope (TEM, HT7700, Japan) was employed to view the sections.

### 2.7 Malondialdehyde and Hydroxyl radical concentration measurement

Malondialdehyde (MDA), a hypothetical marker of oxidant‐mediated lipid peroxidation, was measured by using a commercial kit (Jiancheng BioEngineering Institute, A003‐2‐1, China). Hydroxyl radical (**·**OH), one kind of toxic ROS, was measured by using a commercial kit (Jiancheng BioEngineering Institute, A018‐1‐1, China). The operation was carried out according to the instructions of the kit.

### 2.8 Masson and immunohistochemistry stain

Hearts were harvested, fixed with 4% paraformaldehyde and then paraffin‐embedded. After that, 5‐mm sections were made. Masson staining were employed to assess cardiac fibrosis along with collagen deposition by a Masson stain kit. The whole‐heart fibrosis (fibrosis area/whole heart area) and left ventricular CVF (collagen area/total observed area in multiple random ×20 magnification visual fields) were computed as the ratio of fibrosis area to the overall or left ventricular assessed area quantified with the ImageJ software (V.1.50, Bethesda, USA). Soaking of the sections in 3% H_2_O_2_ was done to stop the endogenous peroxidase activity. Subsequently, the sections were inoculated overnight with anti‐NLRP3 antibody (Novus, NBP2‐12446, USA; 1:200), anti‐α‐SMA (Abcam, ab124964, USA; 1:200) and anti‐Fibronectin (Abcam, ab2413, USA; 1:200) at 4°C for immunohistochemistry. We randomly selected 5 vision fields (×20) for every section with a microscope (Olympus, Tokyo, Japan).

### 2.9 Hydrogen culture medium

In a nutshell, we made a hydrogen‐containing medium by dissolved hydrogen gas into DMEM under 0.4 MPa pressure based on the method described by Ohsawa et al.^8^ Then, the concentration of hydrogen in the culture medium was measured by gas chromatography, ensuring that >0.6 mmol/L.

### 2.10 Isolation and culture of primary cardiomyocytes and cardiac fibroblasts

Cardiomyocytes (CMs) and cardiac fibroblasts (CFs) were harvested from 1‐ to 3‐day‐old neonatal rats. Briefly, rat's heart was quickly removed and 0.04% collagenase II was employed to digest the minced ventricles for six cycles. Cells were harvested and suspended in DMEM medium enriched with 10% FBS. CMs and CFs were collected by differential centrifugation. After 48 hours of culture, BrdU (b5002, sigma Aldrich, United States) was added to the CMs to remove a small amount of residual CFs. CFs were cultured continuously and passaged normally. The second generation was used for experiment.

### 2.11 In vitro experiment protocol

Cells with 75–80% confluence were used for experiments. Prior to all experiments, cells were serum‐starved for 12 h. The CMs were put into a self‐made hypoxia incubator (95% N_2_ and 5% CO_2_) to obtain cell hypoxia model. According to previous experiments, MCC950 was diluted to 10μM with culture medium.^23^

The CMs were divided into five groups: Control group: 10% FBS DMEM; Hypoxia group: 0% FBS DMEM in Hypoxia environment; Hypoxia + H_2_ group: 0% FBS hydrogen culture medium in Hypoxia environment; Hypoxia + MCC950 group: 0% FBS culture medium with 10 μM MCC950 in Hypoxia environment; Hypoxia + MCC950 + H_2_ group: 0% FBS hydrogen culture medium with 10 μM MCC950 in Hypoxia environment.

According to previous experiments, 1 nM Angiotensin II (Ang II) (A118759, Aladdin, USA) was added into the medium to induce CFs proliferation and activation.^24^ The CFs were divided into five groups: Control group: 10% FBS DMEM; Ang II group: 10% FBS DMEM with 1 nM Ang II; Ang II + H_2_ group: 10% FBS hydrogen culture medium with 1nM Ang II; Ang II+MCC950 group: 10% FBS culture medium with 1 nM Ang II and 10 μM MCC950; Ang II+MCC950+H group: 10% FBS hydrogen culture medium with 1 nM Ang II and 10μM MCC950.

### 2.12 Cell viability assay and lactate dehydrogenase release detection

The Cell Counting Kit‐8 (CCK‐8, Abcam, ab228554, USA) was employed to explore cell viability as described by the manufacturer. Lactate dehydrogenase (LDH) release was explored using a commercial kit (Jiancheng BioEngineering Institute, A020‐2–2, China) as documented in the manual provided by the manufacturer to assess cell damage.

### 2.13 Hoechst 33342/PI fluorescent staining

Hoechst 33342/PI double fluorescent staining was employed to assess cell death. CMs experienced hypoxia and the intervention of H_2_ and/or MCC950. Afterwards, Hoechst 33342 staining of the cells in the dark was done at 37℃ for 10 minutes. Subsequently, staining of the cells in 5 μL PI (propidium iodide) was done in the dark at 25℃ for 15 minutes. After that, a confocal laser scanning microscope (FV300, Olympus, Japan) was employed to visualize the cells.

### 2.14 Cell migration assays

Transwell and wound‐healing assays were performed to estimate the migration ability of CFs. In the Transwell assay, CFs (3 × 10^4^ cells/well) were introduced to the upper compartment of a 24‐well cell culture chamber (8 μm pore size, Corning, USA) in serum‐free DMEM with hydrogen and/or Ang II incubation for 24 hours. After that, we fixed the cells with 4% PFA, and then, staining in 0.5% methyl violet solution was done, followed by imaging. In the wound‐healing assay, a sterile 200‐μL pipette tip was employed to scrap the bottom of the monolayer cells when the CFs were grown to confluence in cell plates. Cells were left to migrate freely to the denuded area for 24 hours after treated with hydrogen culture medium and/or Ang II in serum‐free DMEM. The mean linear movement speed of wound edges was determined to reflect the migration relative speed.

### 2.15 Immunofluorescence staining

Immunofluorescence staining was employed for to explore α‐SMA expression in the CFs cells. After treatment, the CFs cells were fixed with 4% paraformaldehyde (PFA) and inoculated overnight with rabbit anti‐α‐SMA (Abcam, USA; 1:200) overnight at 4°. After that, the cells were inoculated for one hour with goat anti‐rabbit antibody (Proteintech, China; 1:300) at 25°C, and then, DAPI counterstaining (Beyotime, China) was performed. A fluorescence microscope (FV300, Olympus, Japan) was employed to visualize the cells. Random discontinuous field of view was selected for photographing, and each group was repeated for 3 times.

### 2.16 RNA extraction, retro transcription and real‐time PCR

Total RNAs from heart tissues were extracted using 1 mL of Trizol reagent (Invitrogen) according to the manufacturer's instructions. cDNA synthesis was performed using the High Capacity cDNA Reverse Transcription Kit (Applied Biosystems, Carlsbad, CA, USA, Cat. no. 4368814) according to the manufacturer's instructions. The SYBR Green PCR Master Mix Kit (Applied Biosystems, Cat. no.4309155) was used to quantify the relative mRNA levels of NLRP3, ASC, Caspase‐1, GSDMD and IL‐1β. Real‐time PCR was performed with the 7500 FAST Real‐Time PCR System (Applied Biosystems) for 40 cycles, with β‐actin serving as internal controls. The sequences of primer pairs are as follows:

NLRP3: Forward, 5′‐TGTTGTCAGGATCTCGCA‐3′ and Reverse, 5′‐AGTGAAGTAAGGCCGGAAT‐3′;
ASC: Forward, 5′‐TGGCTACTGCAACCAGTGTC‐3′ and Reverse, 5′‐ GACCCTGGCAATGAGTGCTT‐3′;
Caspase‐1: Forward, 5′‐TGGAGCTTCAGTCAGGTCCAT‐3′ and Reverse, 5′‐ ACTTGAGGGAACCACTCGGT‐3′;
IL‐1β: Forward, 5′‐GCAGCTTTCGACAGTGAGGA‐3′ and Reverse, 5′‐ CCCAAGTCAAGGGCTTGGAA‐3′;
GSDMD: Forward, 5′‐CAGTGCTCCAGAACCAGTGTC‐3′ and Reverse, 5′‐ ACACGTCATCCCCACGATTC‐3′;
β‐actin: Forward, 5′‐CTAGGCACCAGGGTGTGATG‐3′ and Reverse, 5′‐ AGGTCTCAAACATGATCTGGGT‐3′.

### 2.17 Western blotting analysis

Protein samples were fractionated on a 12% SDS‐PAGE gel and transfer‐embedded onto PVDF membranes. The membranes were inoculated overnight with antibodies of NLRP3 (NBP2‐12446, Novus, USA; 1:200), Caspase‐1 (22915–1‐AP, Santa Cruz Biotechnology, China; 1:1000), GSDMD (sc‐393581, Santa Cruz Biotechnology, USA1:200), IL‐1β (ab9722, Abcam, USA; 1:1000), ASC (sc‐514414, Santa Cruz Biotechnology, USA1:200), Collagen I (14695–1‐AP, Proteintech, China; 1:1000), Collagen III (22734–1‐AP, Proteintech, China; 1:1000), TGF‐β (ab179695, Abcam, USA; 1:1000), α‐SMA (ab124964, Abcam, USA; 1:1000) and β‐actin (sc‐47778, Santa Cruz Biotechnology, USA; 1:1000), respectively, at 4℃. After washing by TBST, the membranes were inoculated at room temperature with horseradish peroxidase‐labelled secondary antibodies (SA00001‐1 and 2, Proteintech, China; 1:2000) for one hour. Afterwards, the ECL reagent was employed to develop the bands. Subsequently, autoradiography was employed to visualize the protein bands and analysed with ImageJ. β‐actin served as the normalization reference.

### 2.18 Caspase‐1 activity assay

The activity of caspase‐1 was measured by using the Caspase‐1 Activity Assay Kit (BC3810, Solarbio, China) in keeping with the manufacturer's instruction. The specimens from tissues and cells were lysed by the lysis buffer. The contents of total proteins were evaluated by Bradford method, and a microplate reader was employed to examine the optical density (OD) values at the wavelength of 405 nm, which was utilized to represent caspase‐1 activity according to the previous study.^25^

### 2.19 Statistical analysis

All the data are given as means ± standard error of mean (SEM). Normality of data distribution was assessed by the Shapiro‐Wilk test prior to the application of parametric tests. To test the significance of the difference between each group, one‐way analysis of variance (ANOVA) with SNK test statistics was applied. *p *< 0.05 defined statistical significance.

## 3 RESULTS

### 3.1 Inhalation of H_2_ improves cardiac function and alleviates left ventricular remodelling in MI rats

**FIGURE 2:**
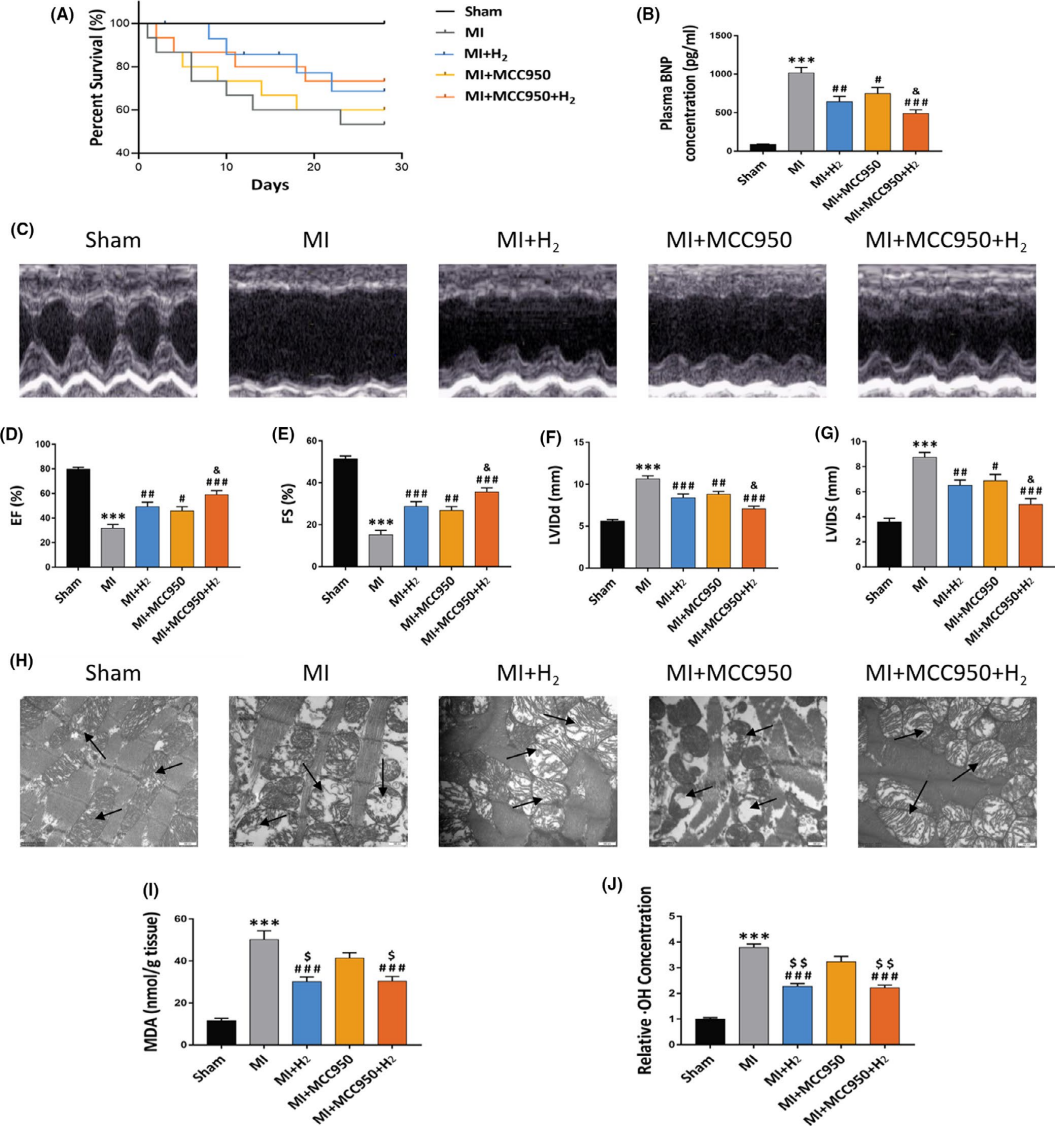
Effects of H_2_ inhalation on cardiac function and myocardial structure changes in rats. (A) Survival curve of rats of each experimental group; (B) the plasma BNP concentration of each group, n = 5 per group; (C) images illustrating echocardiography of rat's heart; (D–G) EF (%); FS (%); LVIDd and LVIDs. (n = 5 per group); (H) TEM images illustrating the cardiomyocytes (×30k magnification, scale bar 100 nm), the arrow indicates the mitochondria of cardiomyocytes; (I) the heart MDA concentration of each group, n = 5 per group; (J) the heart relative ·OH concentration of each group, n = 5 per group. Data are shown by mean ± SEM, ***p < 0.001 vs Sham group. #*p *< 0.05 ##*p *< 0.01 ###*p *< 0.001 vs MI group. &*p *< 0.05 vs MI + MCC950 group; $$$ *p *< 0.001 vs MI + MCC950 group

Studies have shown that inflammasome is involved in myocardial infarction to promote adverse cardiac remodelling. To search the potential mechanism of biological effect of H_2_ on myocardial infarction, we had set MCC950, the selective NLRP3 inflammasome inhibitor, as a positive control group. Twenty‐eight days after myocardial infarction, 15 of 15 rats (100%) in the sham‐operated group survived, while 8 (53%), 11 (73.3%), 9 (60%) and 12 (80%) rats in the MI, MI+H_2_, MI+MCC950 and MI+MCC950+H_2_ groups survived respectively (Figure 2A).

In order to assess the effects of inhalation of H_2_ on cardiac function and left ventricular remodelling in MI rats, plasma brain natriuretic peptide (BNP) concentration, an index reflecting the severity of heart failure and left ventricular function, and echocardiography were measured at 28 days after MI surgery. In contrast with Sham group, BNP concentration in MI group was increased remarkably, while relative to MI group, BNP concentration was substantially decreased in MI+H_2_ group (~36%), MI +MCC950 group (~27%) and MI +MCC950 + H_2_ group (~51%) (Figure 2B).

In addition, we found that ejection fraction (EF), LV end‐systolic dimension (LVIDd), fractional shortening (FS) and LV end‐diastolic dimension (LVIDs), reflecting cardiac function and cardiac remodelling, were remarkably changed in the MI group in contrast with the sham group. Markedly, the decreased LVIDd and LVIDs levels, in addition to increased EF and FS, were found in the MI + H_2_, MI +MCC950 and MI +MCC950 +H_2_ groups in contrast with the MI group at 28 days post‐MI (*p* < 0.05). It is important to note that the MI+MCC950+H_2_ group had additional improvements in cardiac function and decrease in blood BNP concentration relative to the MI+MCC950 group (*p* < 0.05) (Figure 2C‐G). In order to observe the effect of hydrogen inhalation on mitochondrial injury caused by myocardial infarction, the myocardial mitochondrial ultrastructure changes were evaluated by TEM, and the results demonstrated that H_2_ can alleviate the disorganized, as well as damaged mitochondrial architecture in myocardium (myofilament rupture, iliac crest loss, mitochondrial swelling and vacuole formation) induced by myocardial infarction (Figure 2H).

In addition, to better understand the effect of hydrogen gas on ROS level, MDA, a lipid peroxidation product, was selected as a measure of oxidative stress. Moreover, the major toxicity oxygen species (ROS), hydroxyl free radicals (·OH), was chosen to reflect the anti‐oxidative stress effect of hydrogen. The results demonstrated that hydrogen inhalation could significantly reduce the expression of MDA and ·OH in myocardial tissue of rats with myocardial infarction, while MCC950 could not significantly reduce mitochondrial damage and oxidative stress indexes as hydrogen (Figure 2I‐J), which indicated the different mechanisms of regulating NLRP3 inflammasome of hydrogen and MCC950.

### 3.2 Inhalation of H_2_ reduces myocardial fibrosis area and the expression of fibrosis‐related proteins in MI rats

Excess myocardial fibrosis is an important pathological mechanism of heart failure after myocardial infarction. In addition, it also poses developmental difficulties to the treatment of patients with MI after a long time period of myocardial ischaemia. To explore the influence of hydrogen on the cardiac fibrosis, Masson staining was performed to determine the fibrosis area of paraffin‐embedded myocardial tissue sections.

**FIGURE 3:**
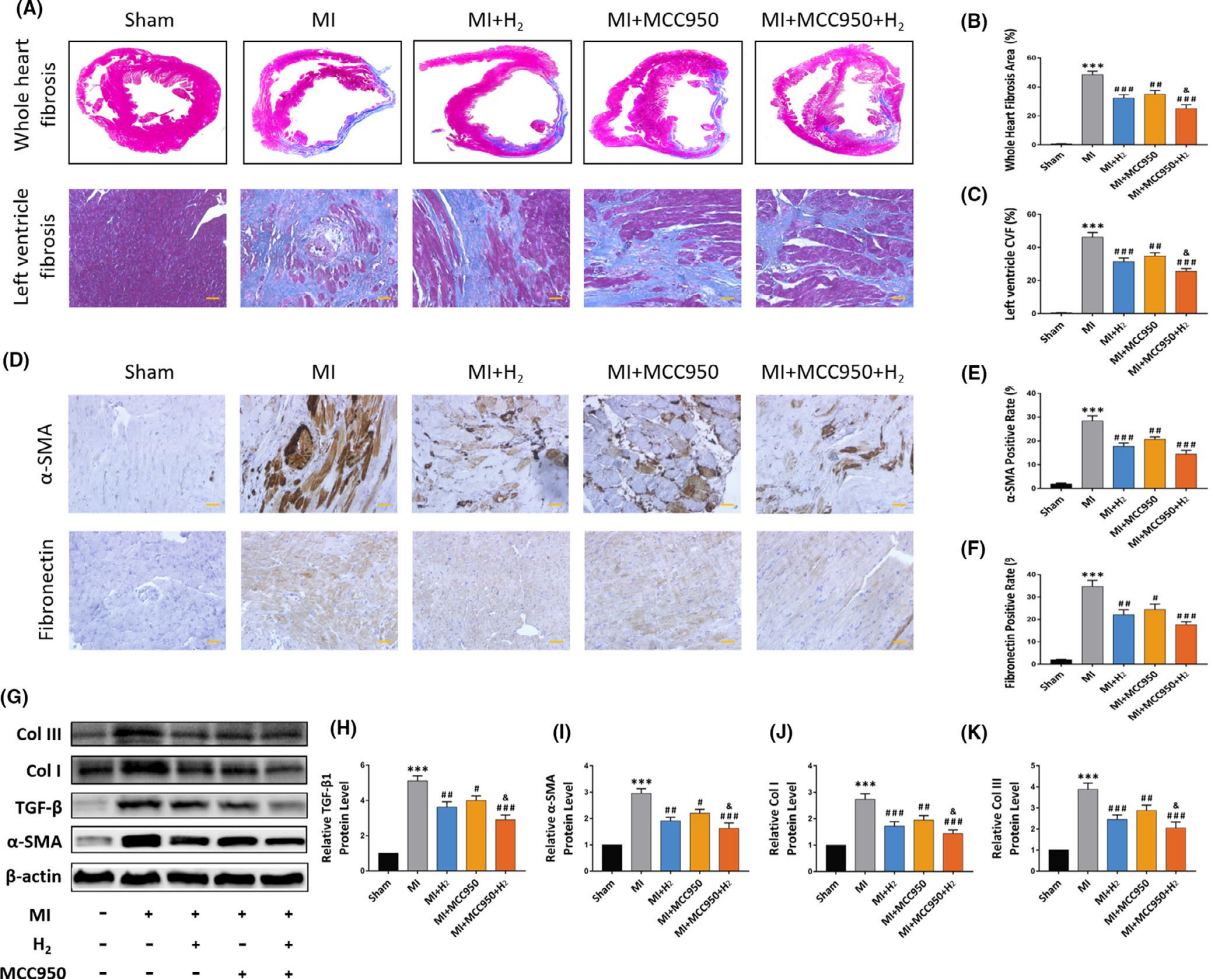
Effects of inhalation of H_2_ on myocardial fibrosis and fibrosis‐related proteins in MI rats. (A) The representative Masson images of rat whole heart and left ventricle; (B) fibrosis area of whole heart sections. n = 5 per group; (C) collagen volume fraction of left ventricle. n = 5 per group; (D) the representative immunohistochemical staining images of α‐SMA and fibronectin expression in each group (×20 magnification, scale bar 50 μm); (E and F) quantification of α‐SMA and Fibronectin positive cells statistical chart, n = 5 per group; (G) the representative Western blot bands of TGF‐β, α‐SMA, Col I and Col III; (H‐K) relative TGF‐β, α‐SMA, Col I and Col III protein level, n = 5. Data are shown by mean ± SEM, ***p < 0.001 vs Sham group; #*p *< 0.05 ##*p *< 0.01 ###*p *< 0.001 vs MI group; &*p *< 0.05 vs MI + MCC950 group

As illustrated in Figure 3A–C, in contrast with MI group, the area of whole heart fibrosis and left ventricle collagen volume fraction (CVF) was reduced by ~27% in MI+H_2_ group and ~21% in MI+MCC950 group respectively (*p*<0.05). Moreover, the area of cardiac fibrosis was substantially reduced by ~47% in MI+MCC950+H_2_ group in contrast with MI group (*p *< 0.05).

To further observe the expression differences of fibrosis related proteins in all experimental groups, immunohistochemistry and Western blot were performed using standard procedures. Consistent with enhanced fibrogenesis, elevated expression of fibrosis‐related proteins including α‐SMA, fibronectin, TGF‐β, Collagen I and Collagen III were detected in MI group in contrast with those in Sham group.

In sharp contrast,the expression of fibrosis‐related proteins was remarkably down‐regulated in the H_2_ inhalation and/or injection of MCC950 group (Figure 3D‐K). Surprisingly, the fibrosis‐linked protein expression was reduced more remarkably in the MI+H_2_+MCC950 group relative to the MI+MCC950 group, indicating H_2_ inhalation combined with MCC950 treatment exhibited better therapeutic effect in alleviating myocardial fibrosis in contrast with MI+MCC950 group (*p* < 0.05). Above all results show that H_2_ inhalation can attenuate cardiac ventricular fibrosis after myocardial infarction.

### 3.3 Inhalation of H_2_ reduces the expression of NLRP3‐mediated pyroptosis‐related protein in MI rats

**FIGURE 4:**
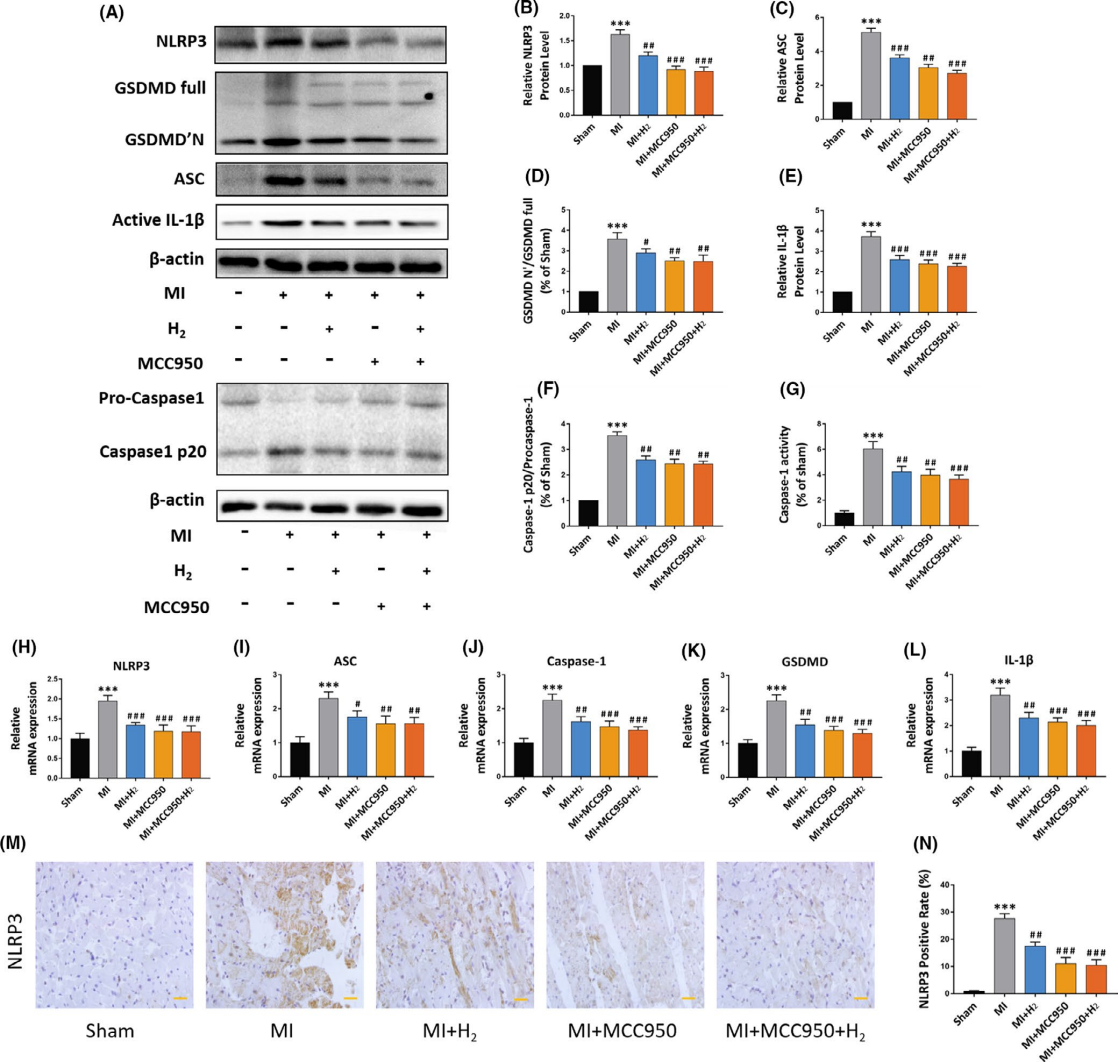
Effect of H_2_ inhalation on NLRP3‐mediated pyroptosis related proteins in MI rats. (A) The representative Western blotting bands of NLRP3, ASC, Caspase‐1, GSDMD and IL‐1β; (B–F) relative NLRP3, GSDMD. IL‐1β, ASC and Caspase‐1 protein level; (G) relative Caspase‐1 activity level, n = 5 per group. (H–L) Relative NLRP3, GSDMD. IL‐1β, ASC and Caspase‐1 mRNA expression, n = 5 per group; (M) images illustrating immunohistochemical staining of NLRP3 expression in each group (×20 magnification, scale bar 50 μm); (N) quantification of NLRP3‐positive cells statistical chart, n = 5 per group. Data are shown by mean ± SEM, ***p < 0.001 vs Sham group; #*p *< 0.05 ##*p *< 0.01 ###*p *< 0.001 vs MI group

Inflammasome plays a critical role in adverse cardiac remodelling after myocardial infarction. To further explore the potential mechanism of biological effect of H_2_ improving cardiac function in rats, we detected NLRP3‐mediated pyroptosis‐associated proteins, which were including NLRP3, ASC, Caspase‐1, GSDMD'N and IL‐1β by Western blot and qRT‐RCR. Western blot and qRT‐PCR data illustrated that H_2_ inhalation remarkably reduced the expression of NLRP3, IL‐1β, ASC, GSDMD'N and Caspase‐1 in myocardial tissue in contrast with MI group, and the therapeutic impact was similar to NLRP3 inhibitor MCC950 group. In addition, we also detected the activity of Caspase‐1, the key molecule of cell pyroptosis. The results showed that caspase‐1 was significantly activated after MI, while hydrogen inhalation and MCC950 could reduce the activity of Caspase‐1 (*p <* 0.05) (Figure 4A‐L).

IHC results further demonstrated that the expression of NLRP3 in MI group was remarkably higher in contrast with other groups. No remarkable difference was found between H_2_ inhalation group and MCC950 treatment group (*p >* 0.05) (Figure 4H‐I). Significantly, the above data supported that the H_2_ could downregulate the expression of pyroptosis‐linked proteins in MI rats.

### 3.4 H_2_ improves cell viability and alleviates pyroptosis in hypoxia cardiomyocytes

In vitro experiment, cardiomyocytes (CMs) were subjected to hypoxia conditions for different durations (1 h, 2 h, 4 h, 8 h and 12 h) to simulate the state of MI injury in rats. We established that NLRP3 expression was highest in 4 hours of hypoxia, with moderate amount of cell death (Fig. S1A,B). Therefore, our follow‐up experiments were conducted 4 hours after induction of hypoxia.

**FIGURE 5:**
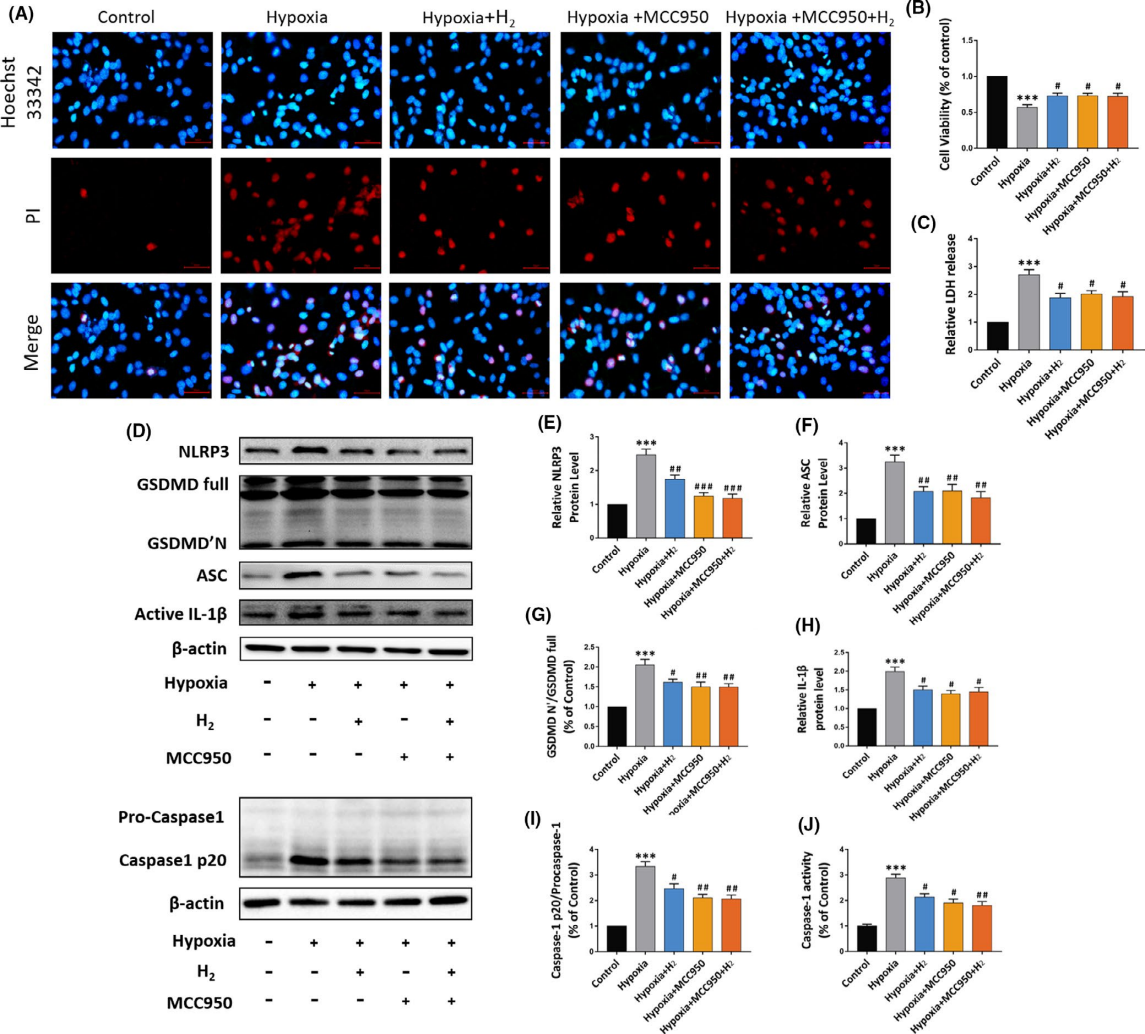
Effect of H_2_ on hypoxia injury and pyroptosis‐related proteins in cardiomyocytes. (A) Representative immunofluorescence image of Hoechst 33342/PI (×20 magnification, scale bar 50 μm); (B) result of CCK‐8 analysis, n = 3; (C) result of LDH release analysis, n = 3; (D) the representative Western blot bands of NLRP3, ASC, Caspase‐1, GSDMD and IL‐1β; (E–I) relative NLRP3, ASC, Caspase‐1, GSDMD and IL‐1β protein level, n = 5; (J) relative Caspase‐1 activity level, n = 5 per group. Data are given as mean ± SEM, ***p < 0.001 vs Control group; #*p *< 0.05 ##*p *< 0.01 ###*p *< 0.001 vs Hypoxia group

In order to validate the effect of H_2_ on pyroptosis at the cellular level, Hoechst 33342/PI double‐fluorescence staining was employed to assess the hypoxia‐induced pyroptosis. Expectedly, the uptake of PI was remarkably increased in the hypoxia group in contrast with that in the control group, whereas the PI uptake was remarkably lowered in the H_2_ and MCC950 treated groups, which further confirmed that H_2_ could reduce the hypoxia‐induced pyroptosis of cardiomyocytes (CMs) (Figure 5A). In addition, CCK‐8 and the release of LDH were used to reflect cardiomyocytes injury. As can be seen from the results in Figure 5B,C, both H_2_ and MCC950 can alleviate the damage of hypoxia to myocardial cells. However, Hypoxia+MCC950+H_2_ group did not show additional better protective effect in CMs in contrast with Hypoxia+H_2_ group or Hypoxia+MCC950 group. Moreover, the effect of hydrogen on hypoxia‐induced pyroptosis of cardiomyocytes was further assessed via Western blotting, and the data demonstrated that the expressions of NLRP3, GSDMD'N, ASC, IL‐1β and Caspase‐1 were remarkably increased in hypoxia group, while the expressions were remarkably decreased in H_2_ and MCC950 groups, which was consistent with previous experiments on hydrogen inhalation in animals (*p *< 0.05) (Figure 5D‐I). In addition, caspase‐1 activity was significantly increased in hypoxia cardiomyocytes; however, hydrogen and MCC950 could reverse the increase of Caspase‐1 activity induced by hypoxia (*p *< 0.05) (Figure 5J).

### 3.5 H_2_ alleviates cardiac fibroblasts activation, migration and collagen synthesis induced by angiotensin II

Pathological myocardial fibrosis is a process characterized by the activation and migration of cardiac fibroblasts (CFs) and an excessive collagen I and III deposition.

**FIGURE 6:**
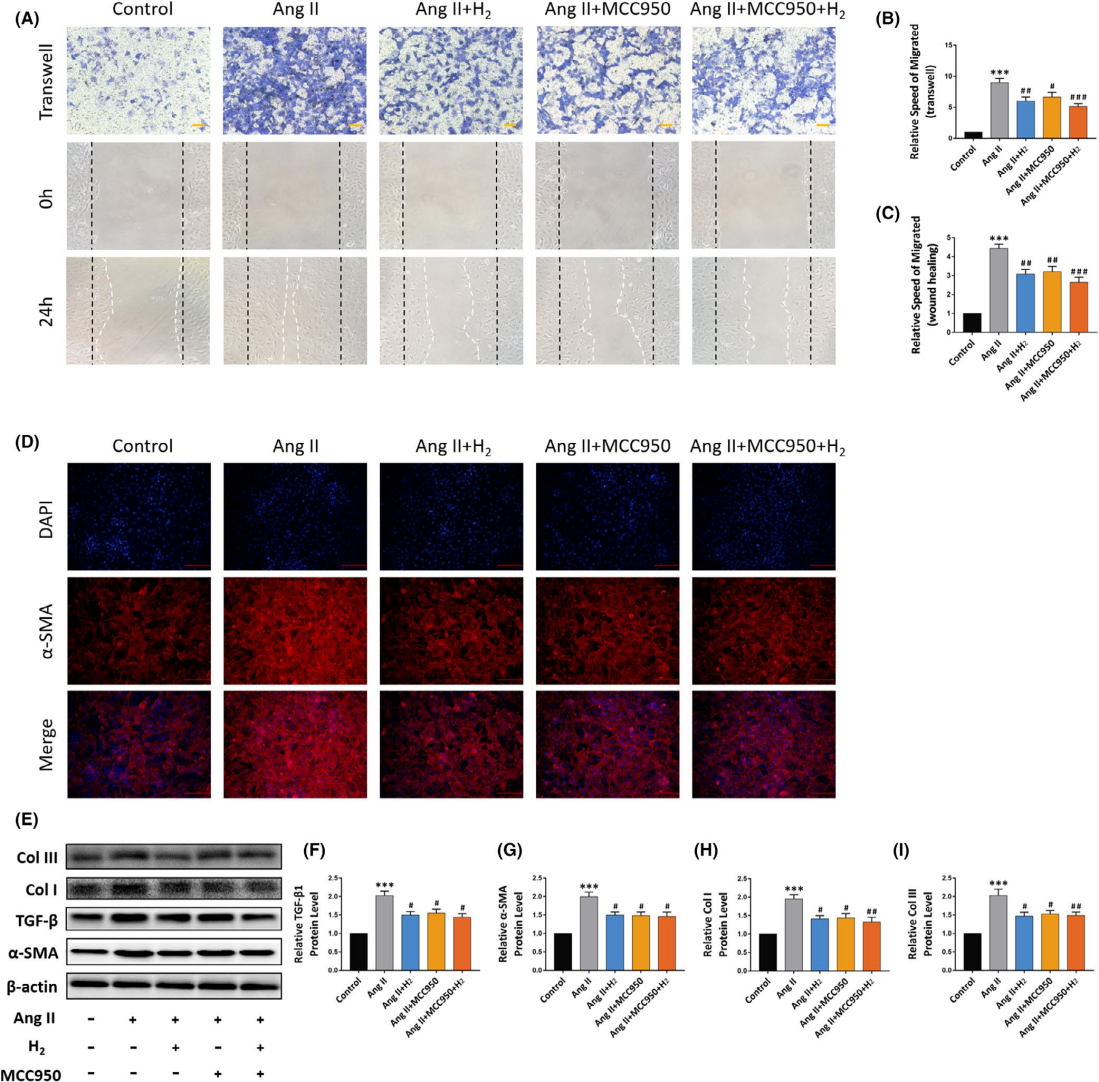
Effect of hydrogen on cell migration and fibrosis‐related proteins in cardiac fibroblasts. (A) The representative Transwell and wound‐healing assay images of CFs. (×10 magnification, scale bar 100 μm); (B and C) relative migration speed of Transwell and wound‐healing assay, n = 3; (D) representative Immunofluorescence image of α‐SMA (×10 magnification, scale bar 100 μm); (E) the representative Western blot bands of TGF‐β, α‐SMA, Col I and Col III; (F‐I) Relative TGF‐β, α‐SMA, Col I and Col III protein level, n = 5. Data are shown by mean ± SEM, ***p < 0.001 vs Sham group. #*p *< 0.05 ##*p *< 0.01 ###*p *< 0.001 vs Ang II group

In addition, Angiotensin II (Ang II) is a critical mediator of cardiac fibrosis. In order to deeply understand the mechanism of H_2_ in attenuating myocardial fibrosis, Transwell and Wound‐healing experiments were performed to observe the effects of hydrogen and MCC950 on CFs migration. Technically, to simulate excessive myocardial fibrosis after MI at the cellular level, we applied Ang II to CFs after careful extraction from rats to promote the activation of CFs. The results showed that migration of CFs was remarkably increased after adding 1 nM Ang II for 24 hours, while the decrease of CFs migration rate was found in Ang II+H_2_ group, Ang II+MCC950 group and Ang II+MCC950+H_2_ group. There was no remarkable difference in cell migration speed among the three groups (Figure 6A‐C). In order to evaluate the activation of CFs induced by Angiotensin II in CFs in different formulation treatment groups, α‐SMA, the key to the activation of cardiac fibroblasts, immunofluorescence staining was performed. Expectedly, the fluorescence intensity of α‐SMA in Ang II+H_2_, Ang II+MCC950 and Ang II+MCC950+H_2_ group was found to be remarkably lower in contrast with those in Ang II group (Figure 6D).

Besides, the expression of fibrosis‐linked proteins TGF‐β, α‐SMA, Col I and Col III in CFs in different treatment groups was explored by Western blot, and it was found that the expression of these proteins in Ang II group was highly upregulated relative to that in Ang II+H_2_, Ang II+MCC950 and Ang II+MCC950+H_2_ groups. There was no remarkable difference in protein expression among these three treatment groups (Figure 6E‐I).

## 4 DISCUSSION

Excessive myocardial fibrosis after myocardial infarction has been proved to be because of the activation of cardiac fibroblasts, which leads to a large amount of collagen deposition through enhanced proliferation and migration.^5^, ^26^ Consequently, the pathological alterations of heart structure ultimately result in refractory heart failure.^27^ Because of the lack of effective therapeutic method to inhibit the proliferation and activation of cardiac fibroblasts, novel treatment to alleviate cardiac remodelling and improve cardiac function is an urgent need.^28^, ^29^

Hydrogen has excellent selective antioxidant capacity and has been proven to have therapeutic effects on multiple diseases.^30^ However, the effect of hydrogen on cardiac remodelling and myocardial fibrosis caused by myocardial infarction has not been adequately studied.

In this study, we investigated for the first time that the effects of hydrogen on myocardial fibrosis and cardiac remodelling in MI rats. We show that inhaling 2% H_2_ for 3 hours daily for 28 days can significantly improve cardiac function and reduce cardiac remodelling in MI rats. Meanwhile, the efficacy of 2% hydrogen also demonstrated through in vitro experiment and the results agreed well with the in vivo data. Specifically, hydrogen can attenuate the death of cardiomyocytes induced by hypoxia, reduce the area of myocardial fibrosis and inhibit the migration and activation of CFs. Moreover, hydrogen also can repress the deposition of collagen I and III, and downregulate the expression of important proteins linked to fibrosis such as TGF‐β, α‐SMA in MI rats. Therefore, H_2_ has great potential to be a new and effective treatment to improve cardiac function and reduce pathological cardiac remodelling and fibrosis after MI. Extraordinarily, unlike rich‐hydrogen saline, which requires liquids volume control, hydrogen inhalation does not involve sodium and water retention, thus more suitable and efficient for clinical application in MI patients.^31^

The mechanism of selective antioxidant effect of hydrogen proposed by Ohsawa et al has been well accepted,^8^ but the antioxidant mechanism of hydrogen is obviously not the only mechanism involved in biological process, suggesting that multiple factors are acting together to exert biological effects.^8^ The diverse biological functions of the hydrogen are still being uncovered. The ability of hydrogen to inhibit apoptosis and regulate metabolism has also been effectively proved.^32^ Notably, oxidative stress is one of the key ways in which NLRP3 is activated.^33^ NLRP3 has been proved to be the most important sensor of aseptic inflammation caused by myocardial infarction.^34^ Activated NLRP3 inflammasome is an important promoter gene of heart failure,^35^ and its downstream inflammatory factor IL‐1β has been used as an important target for the treatment of myocardial infarction in clinical trials.^36^, ^37^ Several main factors play a role in NLRP3 ‘priming’ and ‘activation’ in vivo, such as potassium efflux, lysosomal disruption and oxidative stress induced by mitochondrial damages.^38^

In this experiment, we found that H_2_ could alleviate cell pyroptosis induced by myocardial infarction surgery and hypoxia. To further explore the potential mechanism of biological effect of H_2_ on myocardial infarction, we had set MCC950, the selective NLRP3 inflammasome inhibitor, as a positive control, which has therapeutic effects in a variety of disease models including diabetic arteriosclerotic bacterial inflammation, ischaemia reperfusion injury and so on.^33^ Its primary mechanism of action involves preventing ATP hydrolysis, thus organizing the assembly of inflammatory bodies.^20^, ^21^, ^38^ Our results demonstrated that hydrogen had similar therapeutic effect on cardiac remodelling and fibrosis induced by myocardial infarction as MCC950; however, MCC950 could not reduce oxidative stress indexes as hydrogen, which clearly explains the mechanism of hydrogen that regulate NLRP3 inflammasome by attenuating mitochondrial oxidative stress induced by mitochondrial damages.

Interestingly, when compared with the MCC950 group alone, the combination of H_2_ and MCC950 further improved cardiac function and myocardial fibrosis in rats with myocardial infarction but did not further reduce cell pyroptosis, suggesting that the enhanced therapeutic effect may be largely due to other synergistic biological effects of H_2_, such as anti‐apoptosis^32^ or ·OH scavenging effect.^8^ However, similar phenomenon was not observed in vitro experiments, which may be due to the in vitro experiments could not completely mimic in vivo conditions. Remarkably, our results illustrated that inhibition of NLRP3 inflammasome activation might be a primary mechanism for H_2_ in regulating cardiac remodelling and fibrosis. Studies have shown that with the increase of H_2_ concentration and duration of use, and the treatment efficacy will be enhanced.^39^, ^40^ However, considering the chemical risk of 4% ~75% H_2_ and the related laboratory safety requirements, in this study, rats were inhaled with only 2% H_2_ for 3 hours daily, without setting various subgroups. Future studies on different concentrations and inhalation durations of H_2_ are encouraged while ensuring safety. In addition, based on the therapeutic effect of H_2_ and MCC950 was remarkably enhanced in contrast with MCC950 alone, it is worthwhile to further investigate other potential mechanisms of H_2_ in future studies.

## 5 CONCLUSION

**FIGURE 7:**
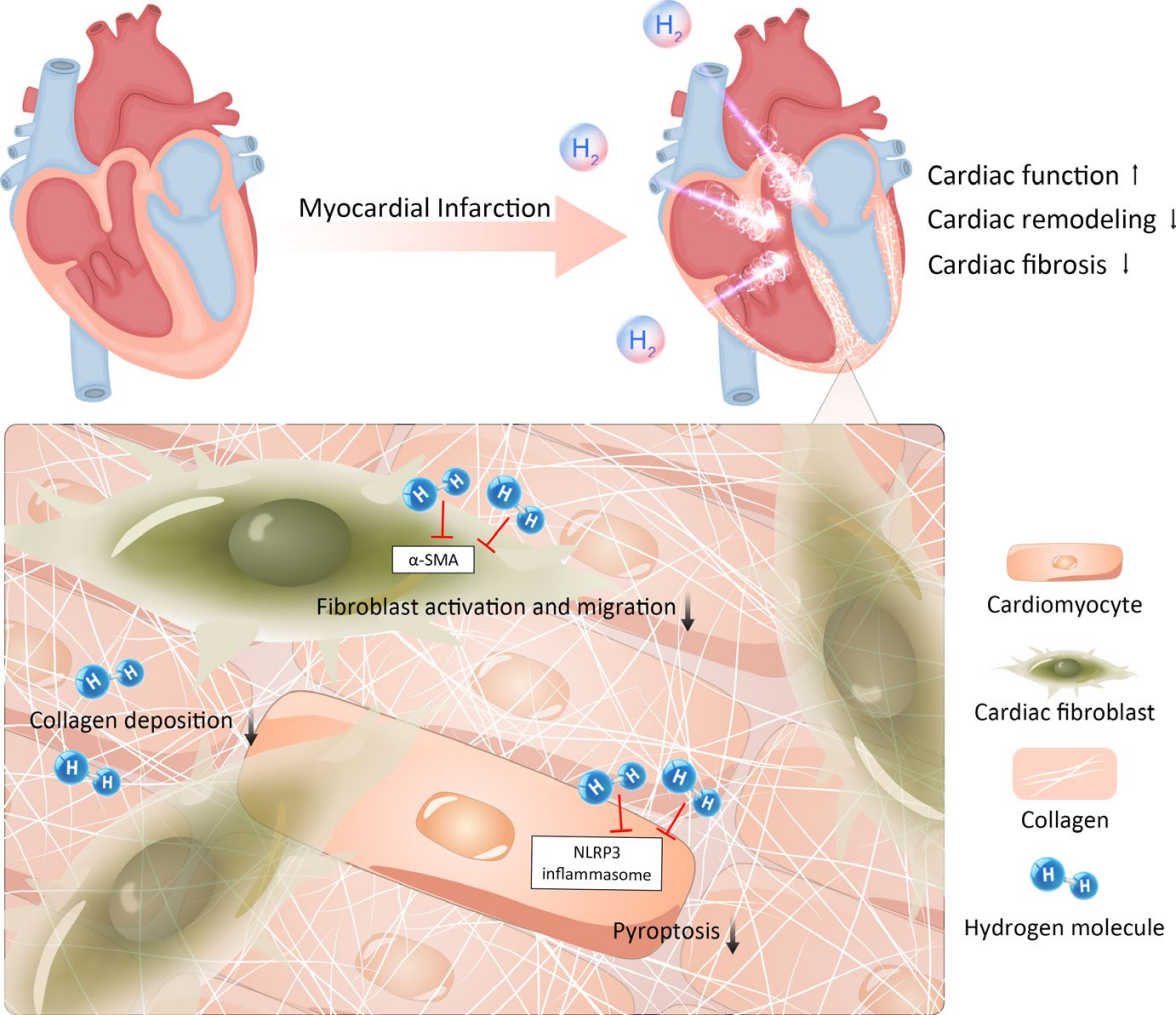
Schematic representation. Schematic diagram of this study: Molecular hydrogen improves cardiac remodelling and fibrosis after myocardial infarction. Specifically, hydrogen further performs pyroptosis by regulating the NLRP3 inflammasome. At the same time, cell viability of cardiomyocytes was enhanced and the migration and activation of cardiac fibroblasts were inhibited. Herein, we found that the anti‐pyroptosis properties of hydrogen have a significant biological effect on cardiac remodelling and myocardial fibrosis after myocardial infarction

Our study suggests that, as a novel gaseous signalling molecules, hydrogen gas can be an effective approach for the clinical treatment of cardiac remodelling and myocardial fibrosis induced by myocardial infarction (Figure 7). In addition, the regulation of NLRP3‐mediated pyroptosis reveals an important biological mechanism of H_2_ in cardiac remodelling and myocardial fibrosis, and provides new insights into the underlying biological effect of hydrogen, which will be helpful for a future feasibility study into other therapeutic applications. Admittedly, because of its good therapeutic effect and high biosecurity, hydrogen has a great potential for clinical translation and is expected to be a novel and effective treatment method for patients with ischaemic heart disease in the future.

## CONFLICT OF INTEREST

None.

## AUTHOR CONTRIBUTIONS

Chaoqun Nie wrote original draft and designed and performed most of the experiments. Rentong Zou and Shuang Pan designed and performed part of the experiments. Rong A, Yunan Gao and Hongxiao Yang analysed and interpreted the data. Juncai Bai, Shuiqing Xi and Xue Wang revised the manuscript. Xiaojian Hong and Wei Yang provide experimental funds, supervised the experiments, revised and approved the manuscript.

## Supporting information

Fig S1

## Data Availability

The experimental data may be obtained from the corresponding author upon reasonable request.
