# Corrigendum

**DOI:** 10.1111/jcmm.17329

**Published:** 2022-06-06

In Nie et al,^1^ the TEM image of the MI +MCC950 + H_2_ group in Figure 2H and the Masson local image for the MI +MCC950 + H_2_ group in Figure 3A cannot be used as representative images. The correct figure is shown below. The authors confirm all results and conclusions of this article remain unchanged.

**FIGURE 2:**
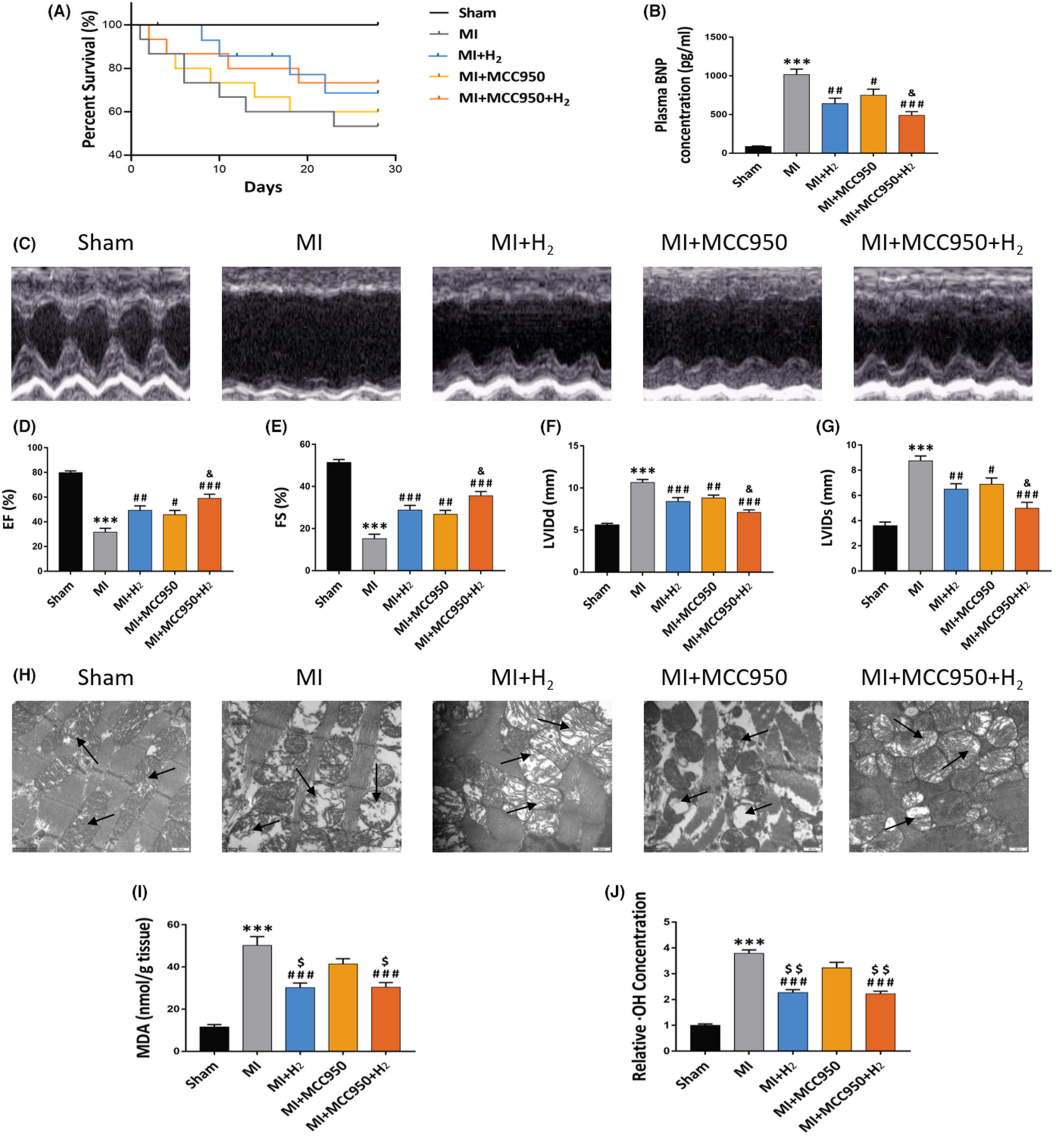
Effects of H_2_ inhalation on cardiac function and myocardial structure changes in rats. (A) Survival curve of rats of each experimental group; (B) the plasma BNP concentration of each group, *n* = 5 per group; (C) images illustrating echocardiography of rat's heart; (D–G) EF (%); FS (%); LVIDd and LVIDs (*n* = 5 per group); (H) TEM images illustrating the cardiomyocytes (×30k magnification, scale bar 100 nm), the arrow indicates the mitochondria of cardiomyocytes; (I) the heart MDA concentration of each group, *n* = 5 per group; (J) the heart relative ·OH concentration of each group, *n* = 5 per group. Data are shown by mean ± SEM, ***p < 0.001 vs Sham group. #*p* < 0.05 ##*p* < 0.01 ###*p* < 0.001 vs MI group. &*p* < 0.05 vs MI + MCC950 group. $$$*p* < 0.001 vs MI + MCC950 group

**FIGURE 3:**
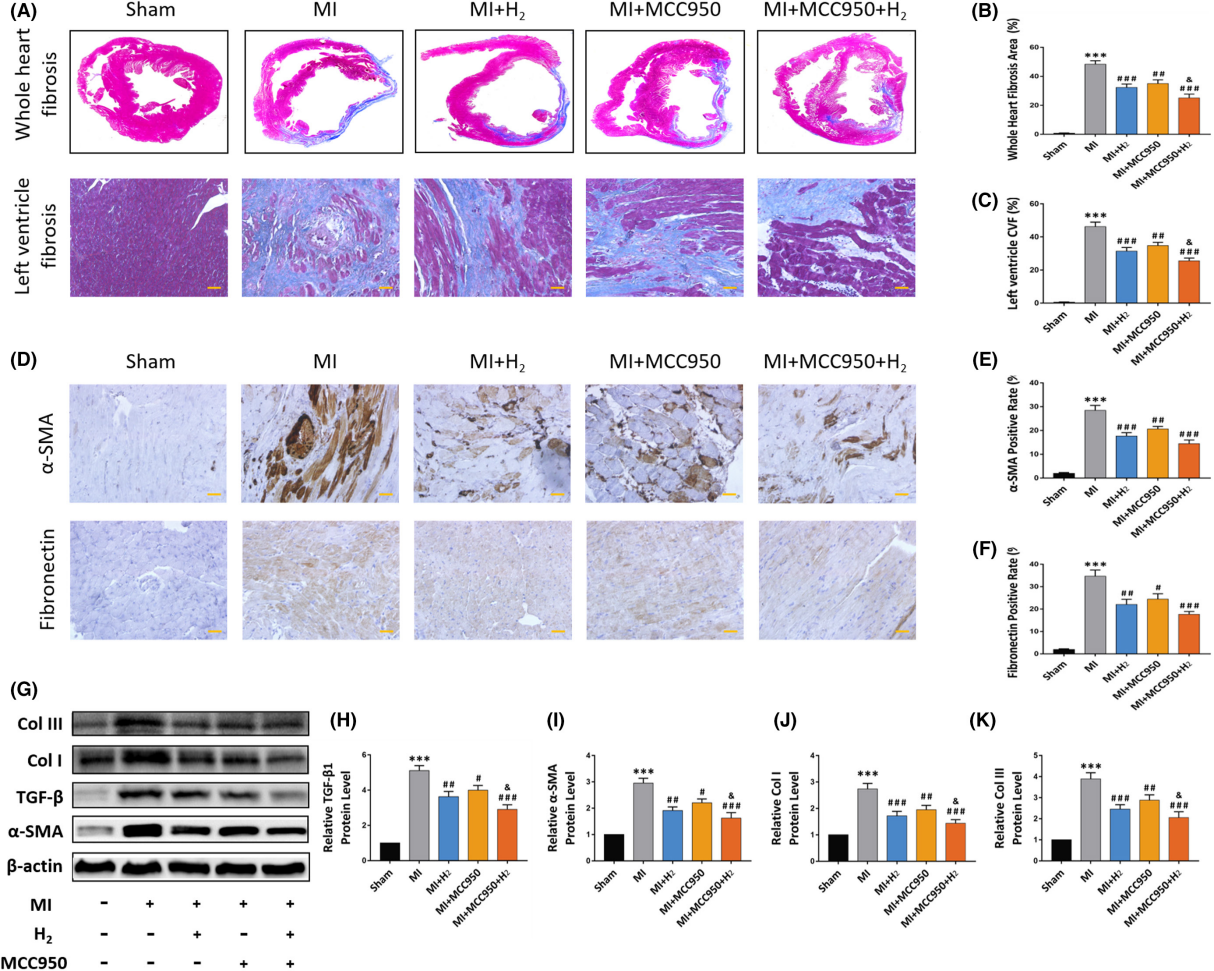
Effects of inhalation of H_2_ on myocardial fibrosis and fibrosis‐related proteins in MI rats. (A) The representative Masson images of rat whole heart and left ventricle; (B) fibrosis area of whole heart sections. *n* = 5 per group; (C) collagen volume fraction of left ventricle. *n* = 5 per group; (D) the representative immunohistochemical staining images of α‐SMA and fibronectin expression in each group (×20 magnification, scale bar 50 μm); (E–F) quantification of α‐SMA and fibronectin‐positive cells statistical chart, *n* = 5 per group; (G) the representative Western blot bands of TGF‐β, α‐SMA, Col I and Col III; (H–K) relative TGF‐β, α‐SMA, Col I and Col III protein level, *n* = 5. Data are shown by mean ± SEM, ***p < 0.001 vs Sham group. #*p* < 0.05 ##*p* < 0.01 ###*p* < 0.001 vs MI group. &*p* < 0.05 vs MI +MCC950 group
