# Supplementary figures and images for: Hydrogen gas inhalation ameliorates cardiac remodelling and fibrosis by regulating NLRP3 inflammasome in myocardial infarction rats
Source: J Cell Mol Med. 2021 Aug 16;25(18):8997–9010. doi: 10.1111/jcmm.16863 (PMC8435412; doi:10.1111/jcmm.16863)

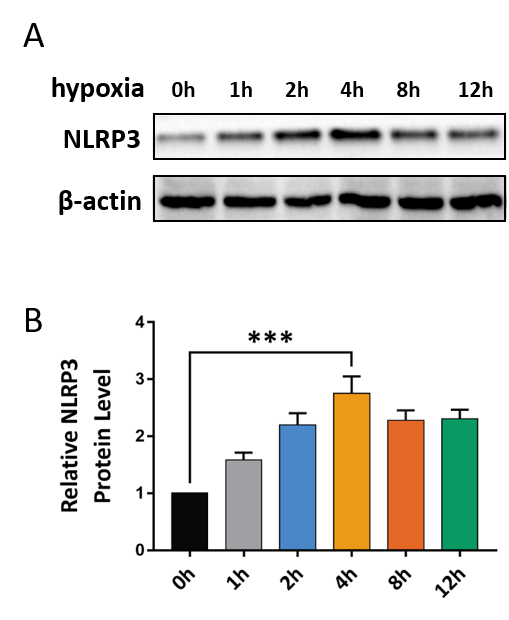

Supplement: Supplementary file 1 — Fig S1 [file JCMM-25-8997-s001.tif]
